# Conditioning period impacts the morphology and proliferative effect of extracellular vesicles derived from rat adipose tissue derived stromal cell

**DOI:** 10.1186/s12951-025-03273-6

**Published:** 2025-03-04

**Authors:** Anton Borger, Maximilian Haertinger, Flavia Millesi, Lorenz Semmler, Paul Supper, Sarah Stadlmayr, Anda Rad, Christine Radtke

**Affiliations:** 1 Department of Plastic, Reconstructive and Aesthetic Surgery, Medical University of Vienna, Währinger Gürtel 18-20, 1090 Vienna, Austria; 2 Austrian Cluster for Tissue Regeneration, Vienna, Austria

**Keywords:** Nerve regeneration, Adipose derived stromal cells, Extracellular vesicles isolation, Exosomes, Proliferation, Schwann cells

## Abstract

A serum-free conditioning period is a crucial step during small extracellular vesicle (sEV) preparation ranging from 12 to 72h. There is a paucity of knowledge about downstream effects of serum-free conditioning on sEVs and the optimal duration of the conditioning period. The aim of this study was to investigate the influence of the serum-free conditioning period on the sEVs derived from primary adipose stromal cells (AdSCs) and their regenerative potential. Primary AdSCs were conditioned in serum-free medium for 72h. Conditioned medium was collected and refreshed every 24h obtaining three fractions, namely sEVs released after 24h (early), 24h to 48h (intermediate) and 48h to 72h (late). After sEV enrichment with ultracentrifugation, the sEV fractions were analyzed by their size, phenotypic expression, and morphology. Proliferation assays of primary Schwann cells after treatment with sEVs were performed. Particles meeting criteria to be classified as sEVs were detected in all fractions. However, sEVs differed by their size and phenotypic expression. A long conditioning period led to a heterogenous population of larger sEVs and increased protein per particle ratio. Moreover, the expression of tetraspanines was affected. Lastly, the proliferative effect of sEVs on Schwann cells decreased with increasing conditioning period. In conclusion, particles meeting the criteria of EVs are released by primary AdSCs over 72h under serum free conditioning. Nonetheless, they significantly differ in their proliferative effect on Schwann cells cultures.

**Graphical Abstract:**

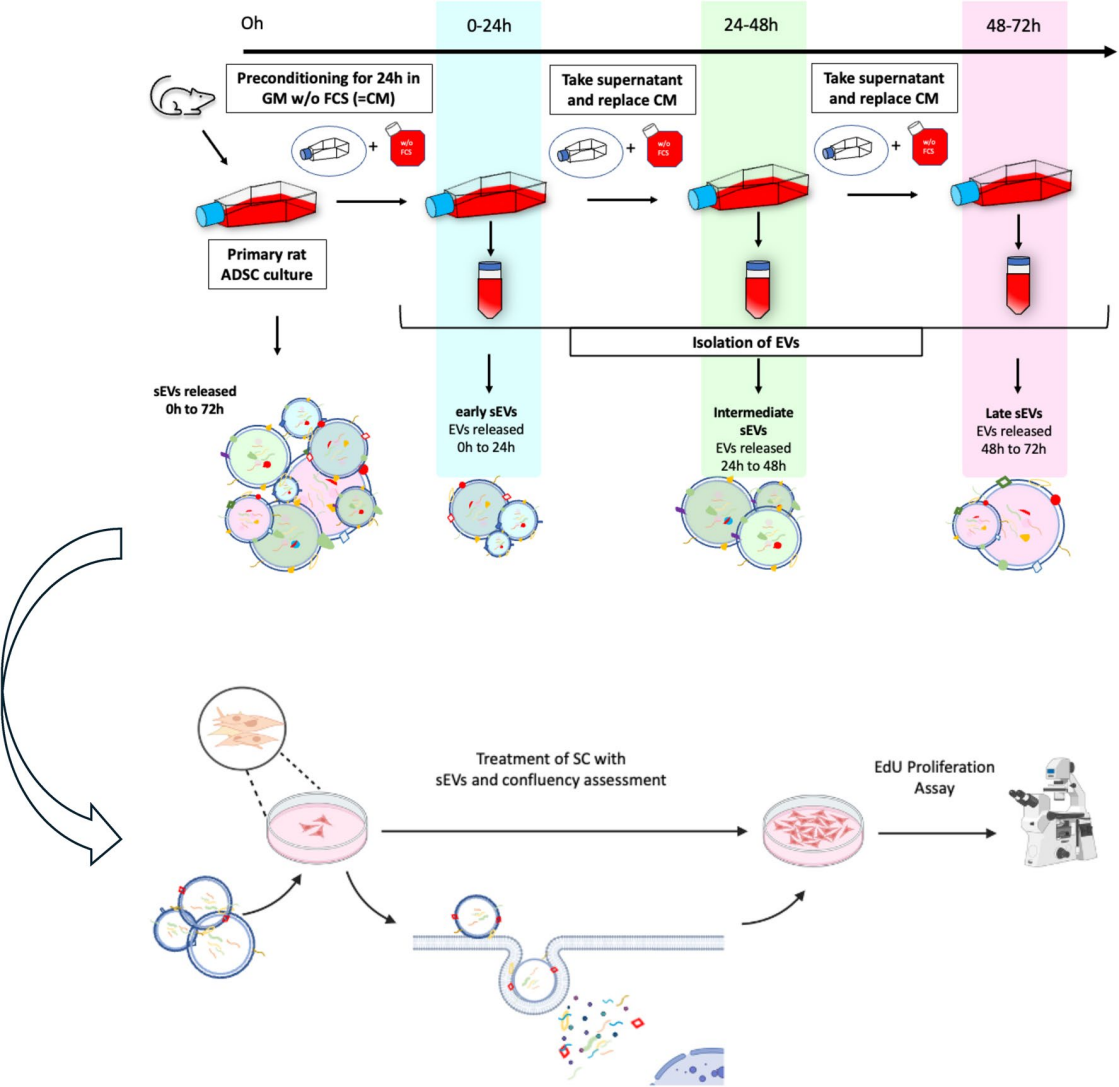

**Supplementary Information:**

The online version contains supplementary material available at 10.1186/s12951-025-03273-6.

## Background

Over the past decade, extracellular vesicles (EVs) have gained broad interest in diverse fields of research. In regenerative medicine, they attracted significant interest as a cell-free therapeutic approach. sEVs are highly biologically active by conveying proteins, nucleic acidsand other molecules to recipient cells influencing their behavior of [1, 2]. sEVs derived from mesenchymal stromal cells (MSC) have attached much recent interest as a potential cell-free therapeutic alternative to their origin cells [3, 4]. Numerous studies demonstrated that sEVs isolated from MSC cultures were able to increase the proliferation of a plethora of cell types [5–7]. A highly promising approach for clinical application arose from the presence of multipotential stromal cells in adipose tissue, which can be obtained on a large scale by liposuction [8]. sEVs isolated from these primary adipose tissue-derived stromal cells (AdSCs) can be reapplied to the patients in an autologous manner enhancing the regeneration of various tissues [9–13].

Cell culture medium is an important component in culture preparations, but often contains serum which can contain sEVs. Thus, to study functional effects of sEVs generated from specific cells, deprivation of serum products in culture medium is necessary due to the occurrence of EVs and co-isolated proteins in serum [14]. However, serum is a widespread standard component of culture media [15], but there is ongoing research for development of serum-free alternatives [5, 16]. Approaches include EV-depleted serum as well as a number of commercial EV-free products. However, a number of recent studies indicate that some extracellular vesicles persist in extracellular vesicle depleted culture media [14, 17–20]. Thus, an influence on functional results of even some contamination from serum vesicles cannot be fully excluded. According to recent guidelines, the declaration of components of media should be included in documentation [21]. Despite the ongoing development of serum replacements, the most common medium for sEV production is serum free as surveyed by Royo et al. [6], which may be explained by the aforementioned shortcomings [20, 21]. The duration of conditioning period found in the literature demonstrates a high variance ranging from overnight to 24 h [9, 13, 22], 48 h [6, 11, 23, 24] and up to 72h [25]. Researchers working with EVs are confronted with the dilemma of short conditioning periods with a high viability of cells and a low yield of EVs, and long conditioning periods achieving higher yields of EVs but a low viability of the cells. This methodical inconsistency leads to a lack of reproducibility and comparability of results.

The aim of this study was to investigate the downstream time-dependent effects of serum-free conditioning on AdSCs and their released sEVs, and the effect the sEVs have on Schwann cell proliferation. By collecting and replacing the conditioned medium every 24 h, we fractioned the sEVs released over 72h by time windows and obtained three distinct sEVs population. Our results shed light on the time-dependent release of sEVs under serum-free culture. The pro-regenerative capacities of these sEVs that could potentially have a crucial role in future clinical applications were investigated via proliferation assays with primary Schwann cells (SC).

## Material and methods

### Animals

Sciatic nerves and intraabdominal adipose tissue were obtained from adolescent male Sprague Dawley rats. In accordance with Austrian Animal Testing Law (TVG 2012, §2, 1.c.) and Article 3 of the Directive 2010/63/EU of The European Parliament and of the Council on the Protection of Animals Used for Scientific Purposes, tissue harvest was obtained from euthanized animals.

### Isolation and culture of primary rat AdSCs

As previously described [13], approximately 5 ml of retroperitoneal, perirenal and gonadal adipose tissue was harvested from 15 euthanized male animals [8, 26]. In brief, 5 to 7ml of whole harvested adipose tissue was minced, enzymatically digested with collagenase type I (Merck, Germany), and centrifuged with 300xg for 5 min at room temperature and seeded in T25cm^2^ flasks. Subsequently, AdSCs were cultured under standard culture conditions (37°C, 95% humidity, 5% CO_2_) in T175cm^2^ flasks. Culture medium was composed of Dulbecco’s Modified Eagle Medium (DMEM) with 4.5g/l D-glucose (Gibco, Thermo Fisher, USA), 10% fetal calf serum (FCS, Linaris, Germany), 1% penicillin/streptomycin (P/S, Gibco, Thermo Fisher, USA), 2ng/ml recombinant human FGF basic (Pepro Tech, United Kingdom). At a confluency of 60–70% AdSCs were either passaged or conditioned for EV isolation.

### Characterization of AdSCs

AdSCs were characterized by immunophenotype and their multilineage differentiation potential [27]. Cell surface antigens were assessed by immunostainings with antibodies against CD73, CD90, and CD105, as previously described [13]. All primary, and secondary antibodies as well as their dilutions are listed in supp. Table 1.

To assess multilineage differentiation potential,AdSCs in passage three (p3) were cultured in osteogenesis, adipogenesis and chondrogenesis inducing differentiation kits acquired from Promocell (Germany) according to the manufacturer’s instructions. The adipocytes, osteocytes, and chondrocytes were stained with Oil Red O, Alizarin Red, and Alcian blue, respectively.

### Viability assays with MTT conversion

The viability and metabolic activity of AdSCs under serum-free conditions were assessed via MTT assay (CellTiter 96® Non-Radioactive Cell Proliferation Assay, Promega, Germany). AdSCs from three donors were seeded with a density of 5,000/cm^2^ in flat-bottom 96-well-plates (Greiner, Austria) in triplicates and cultured until they reached a confluency of 60–70%. The growth medium was replaced with serum-free growth medium for 24h, 48h, or 72h. As time-matched controls, AdSCs were cultured in fresh growth medium. The MTT assay was performed according to the manufacturer’s instructions. Absorbance was measured at 570nm with a reference wavelength of 650nm. The curve was normalized with AdSCs at day zero (d0) for 100% viability and AdSCs exterminated with 70% ethanol as negative control, respectively.

### Preparation and Enrichment of sEVs

**Fig. 1:**
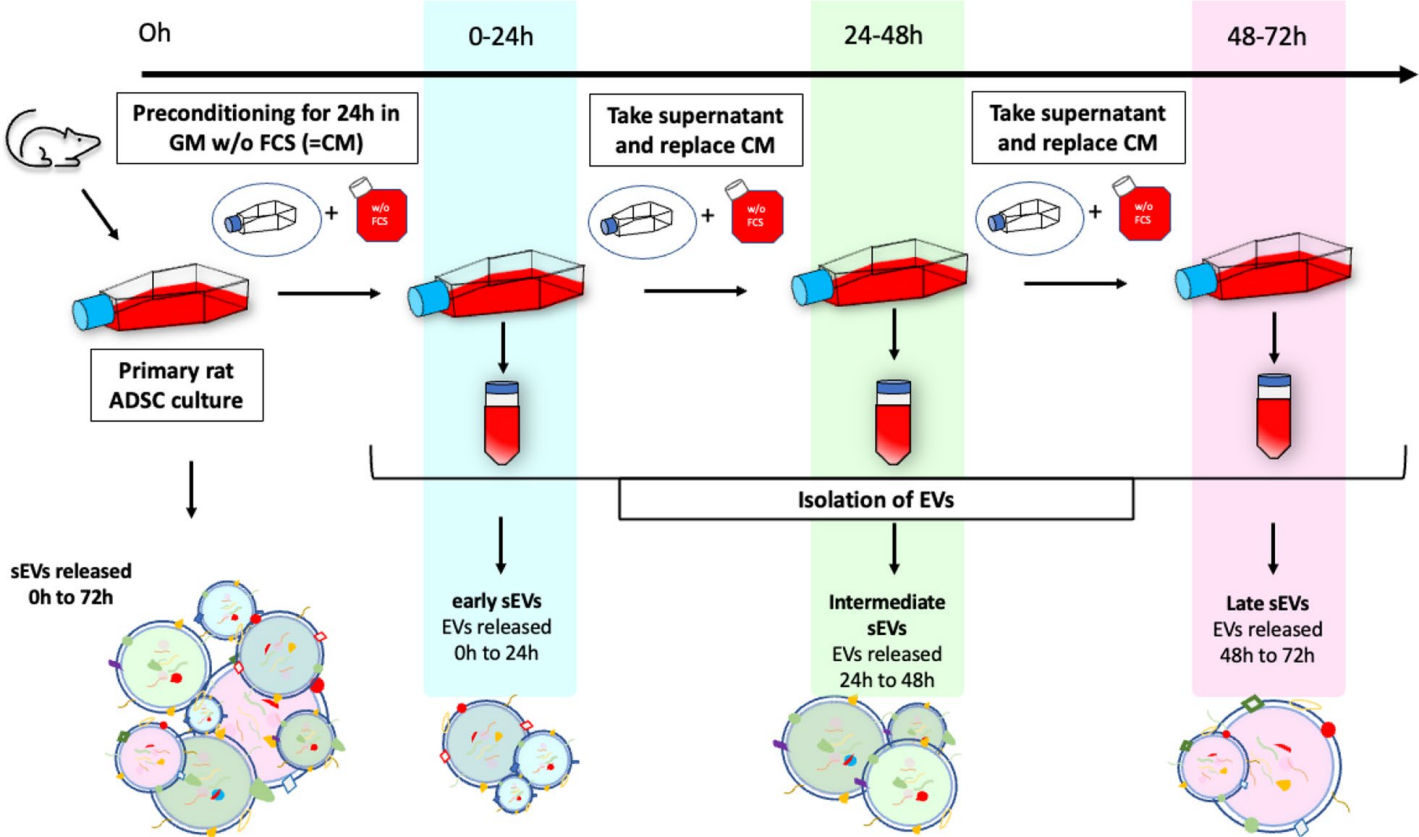
Conditioning and Isolation of sEVs released from primary rat AdSCs in three time-windows. After reaching a confluency of 60–70%, AdSCs in p2 and p3 were conditioned with serum-free growth medium for 24h. Each 24h of conditioning period the medium was collected, and medium was replaced. This step was repeated twice thus obtaining three fractions– 0h to 24h (early); 24h-48h (intermediate); 48h-72h (late). Isolation was performed with differential centrifugation including one filtration step through a 200nm PVDF filter. Pellets containing the three sEV fractions were suspended in the respective media depending on their use and stored at − 80 °C

At a confluency of 60–70%, AdSCs in p2 to p3 cultured in T175cm2 flasks (Greiner Bio-One International GmbH, Austria) were washed twice with Hanks' balanced salt solution (HBSS, Lonza, Basel, Switzerland) and the growth medium was replaced with 20ml FCS-free growth medium. The conditioned medium was collected and replaced after a washing step with HBBS three times every 24h. Thus, we obtained conditioned medium containing sEVs released in three subsequent time windows, which were defined as early (0 to 24h), intermediate (IM) (24 to 48h) and late (48 to 72h) sEVs, as presented in Fig. 1. Conditioned medium was further processed by differential centrifugation steps. [28] Large cell contaminants debris and larger vesicles were depleted at 300xg for 10min at 4°C, 2,000xg for 20min at 4°C followed by a filtration step through a Durapore PVDF (Polyvinylidene Fluoride) 0,22µm filter (Merck, Germany). sEV enrichment was performed by two repeated steps of ultracentrifugation at 100′000 × g for 1,5h at 4°C in a fixed angle rotor (T-865; k-factor_max_ = 51.7; Thermo Fisher Scientific, USA) [28]. After the first ultracentrifugation step the supernatant was withdrawn. Pellets from 40ml of conditioned medium were pooled and resuspended in fresh ice-cold phosphate buffered saline (PBS) without Mg^2+^ and Ca^2+^ (Gibco, Waltham, USA). Subsequently, the second ultracentrifugation step was performed. The supernatant was withdrawn again, and the obtained pellet was resuspended in 100µl ice-cold PBS. Aliquots were stored at -80°C in protein low-binding tubes (Eppendorf AG, Germany) until further use [29]. All specifications of preparation and characterization results of EVs are given in Table 1 and Table 2.

**Table 1:** Specifications of experimental setups according to MISEV 2018,2023

Pre-processing variables	
Isolation source	Conditioned medium from cell culture
Cells	AdSCs
Provenance	Primary cells
Time of culture and confluency	3–4 days, 70–80% confluency
Culture and EV isolation conditions	
Passage	2–3
Culture volume	20ml
Culture vessel	T175cm^2^ flasks
Surface coatings	none
Culture conditions	5% CO_2,_ 37°C, 95% H_2_O
Conditioning medium	DMEM (4.5mg/l D-glucose), 1% P/S, 2ng/ml b-FGF
Collection method	Pipetting from flasks
Isolation	
Differential centrifugation with filtration step	300xg 10 min 4°C 2,000xg 20 min 4°C 200nm PVDF Filter 2 × 100,000xg 1,5h, 4°C Resuspension volume 100µl of PBS
Storage conditions	
	− 80°C in PBS and low protein binding Eppendorf tubes

AdSCs: Adipose tissue derived stromal cells, b-FGF: basal- Fibroblast growth factor, DMEM: Dulbecco’s modified eagle medium, IM: intermediate, PBS: Phosphate-buffered saline, PVDF: Polyvinylidene Fluoride

**Table 2:** Characteristics of Extracellular vesicles according to MISEV 2018, 2023

Characterization			
Quantification	Variable	Results	Method
	Particle number [10^9^]/ml	Early: 6.42 ± 1.46 IM: 4.08 ± 1.75 Late: 3.01 ± 1.83	Zetaview NTA (Fluorescence mode)
	Particle size median [nm]	Early: 97.36 ± 2.39 IM: 110.8 ± 8.96 Late: 118.1 ± 4.40	Zetaview NTA (Fluorescence mode)
	Amount of Protein [µg]	Early: 3.76 ± 1.19 IM: 2.86 ± 0.65 Late: 2.74 ± 1.30	Micro BCA assay with sonification step
	Particle/protein ratio [10^8]^	Early: 10.0 ± 5.07 IM: 7.55 ± 3.30 Late: 6.59 ± 2.93	Micro BCA assay with sonification step vs. NTA results
Phenotype	CD9, CD63 CD81, CD90	Present in all groups	Amnis Imagestream Isx
	Calnexin [%]	Early: 4.34 ± 5.36 IM: 4.45 ± 5.13 Late: 10.84 ± 10.55	Amnis Imagestream Isx
Single Vesicle	bilayer membrane	present in all groups	Cryo-EM
	Size		AFM

AFM: Atomic force microscopy, BCA: Bicinchoninic acid assay, EM: Electron microscopy, IM: intermediate, NTA: Nanotracking Analysis

### Cryo electron microscopy

For cryo-TEM imaging of extracellular vesicles from one donor, Quantifoil copper 200 mesh R2/2 holy carbon grids (Quantifoil, Germany) were glow discharged for 360 s at 25 mA using a BalTec SCD050 sputter coater (Balzers, Liechtenstein) and loaded into a Leica EM GP (Leica Microsystems, Austria) grid plunger. The conditions of the climate chamber were set to 4°C and 80% relative humidity. 4 μl of the sample were applied onto the carbon side of the grid and front‐side blotted for 1s, 2s or 4s using the instrument's sensor function and Whatman filter paper #1 (Whatman, UK). To achieve instant vitrification, the sample was plunge‐frozen in liquid ethane at − 184°C. Images of the vitrified samples were collected with a 200kV Glacios cryo-transmission electron microscope (ThermoFisher Scientific, US) equipped with a X-FEG and a Falcon3 direct electron detector (ThermoFisher Scientific, US). Digital images were recorded using the SerialEM data acquisition software (University of Colorado, Boulder, US). The recording was done in linear mode of the Falcon3 camera at a magnification of 150,000x (pixel size: 0.98 Å, defocus: − 2 μm, total electron dose:60e-/Å2).

### Atomic force microscopy

In order to prepare samples for atomic force microscopy (AFM) measurements, aliquots from one donor of frozen sEVs (early, intermediate, and late) were thawed at room temperature. Subsequently, 10µL of the suspension were transferred to freshly cleaved mica sheets (V1 quality, Electron Microscopy Sciences, Hatfield, USA) and dried overnight at room temperature in a dust-free container. On the next day, sEVs were carefully rinsed with distilled water and air dried for further 2h. Arbitrarily selected areas of sEVs on the mica sheet were imaged in a semi-contact mode via a multimode atomic force microscope (Ntegra Aura, NT-MDT) under ambient conditions. The micrographs were acquired using tapping mode cantilevers (NSG30, NT-MDT) with an end radius of around 6nm and a force constant of 40N/m at a scan rate of 0.2Hz. Post-processing of the AFM micrographs was performed via the software Gwyddion (version 2.60, GNU General Public License). Thereby the background was corrected, and the rows were aligned by applying a second-degree polynomial function. Subsequently, artifacts were removed by step line correction and adjustment of heigh range was performed.

### Nanoparticle tracking analysis (NTA) in fluorescence mode

Size and concentration estimate of sEV fractions was determined by Zetaview PMX-120 (Particle Metrix GmbH, Germany) equipped with a 488nm laser in fluorescence mode. To distinguish phospholipid bilayered particles from other particles, sEVs from five donors were stained with Cell Mask Green ™ Plasma Membrane Stain (CMG, Invitrogen, USA, 1:500) for 20min at 37 °C [30]. Aliquots of 20µl of CMG-labeled EVs isolates were diluted to 1ml and syringed to the device. Measurements were performed at eleven positions with three cycles in fluorescence mode with the following parameters: sensitivity: 85–90, shutter: 50, frame rate: 30, brightness: 30, min area: 10, max area: 9000. The temperature was set to 27°C. Subsequently, measurements in scatter mode were performed with the given setup: sensitivity: 70, shutter: 60, frame rate: 30, brightness: 30, min area: 10, max area: 9000. To account for background signals and staining artifacts, several controls such as CMG in PBS, PBS only, and unstained EV isolates were analyzed.

### Protein measurement

Following enrichment by ultracentrifugation, sEV-containing pellets from five donors were resuspended in 150µl of ice-cold 10 × radioimmunoprecipitation Assay buffer (RIPA buffer) (1:10) supplemented with Halt Protease Inhibitor Cocktail EDTA-free (1:100), (both Thermo Scientific, USA) diluted in ultrapure Milli-Q plus water (Merck Millipore, Germany). To amplify the protein recovery, sEVs were additionally sonicated for 90s (pulse:30%, amplitude:60%) with UP200ST ultrasonic homogenizer (Hielscher Ultrasonic GmbH, Germany). After incubating for 20min at 4°C the aliquots were stored at -80°C. The amount of protein was determined with Micro BCA™ Protein assay (Thermo Scientific, USA) in 96-well microplates following the manufacturer’s protocol for microplate procedure. The absorbance was measured at 562nm according to manufacturer’s recommendation.

### Immunophenotypic characterization of sEVs

Immunophenotypic characterization of sEVs from five donors was performed by imaging flow cytometry (Amnis ImageStream^x^ Mark II Flow Cytometer, ISx, EMD Millipore, USA). Prior to staining, all antibodies were centrifuged at 18,000xg for 5min at 4°C to deplete aggregates. Approximately 5 × 10^6^ sEVs were incubated with CD9-AF488, CD63-AF594, CD81-VioBlue, Calnexin-APC 647 and CMG for 1h at room temperature. The antibodies and concentrations are listed in supp. Table 1. For lysis control, sEVs were lysed with 10 × RIPA buffer for 15min at room temperature prior to staining. Further, negative controls such as unstained EVs and antibodies in PBS only were analyzed. After incubation all samples were diluted to 100µl with PBS. 10µl of stained probes were assessed by ISx. Gating strategy is listed in supp. Figure 1. Lasers were set as follows: 400nm at 120mW, 488nm at 200 mW, 561nm at 200mW, 642nm at 150mW, and SSC at 1mW, magnification at 60x, slow flow rate. Acquisition data was processed with IDEAS 6.0 analysis software (Amnis Cooperation, Seattle, USA).

### Isolation and characterization of primary Schwann cells

Schwann cells (SC) were isolated from dissected sciatic nerves from five adolescent male Sprague Dawley rats as previously described [6, 31]. Fascicles were mechanically pulled with forceps and cut into approximately 0.5cm long pieces. The fascicles were enzymatically digested overnight with collagenase Type IV and dispase II.The pellet was resuspended in SC growth medium and subsequentially seeded in precoated 6-wells with 0.01% poly-L-lysine hydrobromide (PLL, Sigma-Aldrich, USA) and 5 μg/ml laminin (Sigma-Aldrich, USA) followed by a culturing under common culture conditions. SCs enrichment steps were performed by taking advantage of distinct adhesion behavior of SCs and fibroblasts. Purity and immunophenotype of SCs was assessed by immunocytochemistry with common SC markers S-100 and Sox-10, CD90 as a marker for co-isolated nerve-derived fibroblasts as well as vimentin for cytoskeleton and DAPI for nuclei. All materials are displayed in supp. Table 1. Fluorescence pictures were obtained with the Nikon Eclipse Ti (Nikon Instruments Inc., Japan).

### Proliferation experiment

The SCs from five donors cultured in 8 wells (Ibidi GmbH, Graefelfing, Germany) were treated with 5 × 10^8^ sEVs, determined in fluorescence mode by NTA, either from early, intermediate, or late group. Control cells were cultured in growth medium with PBS as vehicle control. Phase contrast micrographs of each well at same spot were obtained at the beginning (0h), 24h, and 48h after the begin of experiment. Image processing was performed with ImageJ2 (version 2.3.0) [32, 33] by a conversion to 8-bit format, adjusting the brightness and levels to eliminate the background signals, and further processing by converting to mask function (supp. Figure 5). Finally, the area covered by cells was calculated. The overgrown area was used for the calculation of increase of confluency. In addition, after 48h of coincubation, 10 μM of 5-ethynyl-2'deoxyuridine (EdU, Click-it™ EdU Cell Proliferation Kit, Invitrogen, USA) was added and incubated for 2h. Subsequently, cells were fixed with 4,5% formaldehyde and in accordance with the manufacturer’s manual, EdU was labeled with Alexa Fluor 555. Afterwards, cells were stained with SOX-10, and DAPI (supp. Table 1).

### Statistical analysis

All statistical analyses were performed by GraphPad Prism 9.0.0 (GraphPad Software, USA). If not displayed, all values are given in arithmetic means with standard deviation. The number of replicates is given in the respective methods section. Tests for significance were performed with two-way ANOVA followed by a Tukey post-hoc correction in case of multiple comparisons. Principal component analysis (PCA) was performed based on eigenvalues, by applying the “Kaiser rule” using values bigger than 1. A significance was set at p ≤ 0.05.

## Results

Primary AdSCs cultures were characterized by morphology, viability immunophenotype, and their differential potential. [27] The presence of AdSCs was demonstrated by plastic adherence, their characteristic spindle shape, expression of CD73^+^, CD90^+^ and CD105^+^ (supp. Figure 2). Further, their multipotency was demonstrated by chondrogenic, osteogenic, and adipogenic differentiation (supp. Figure 3).

### Serum-free culture conditions result in decreasing viability of primary AdSCs depending on time

**Fig. 2:**
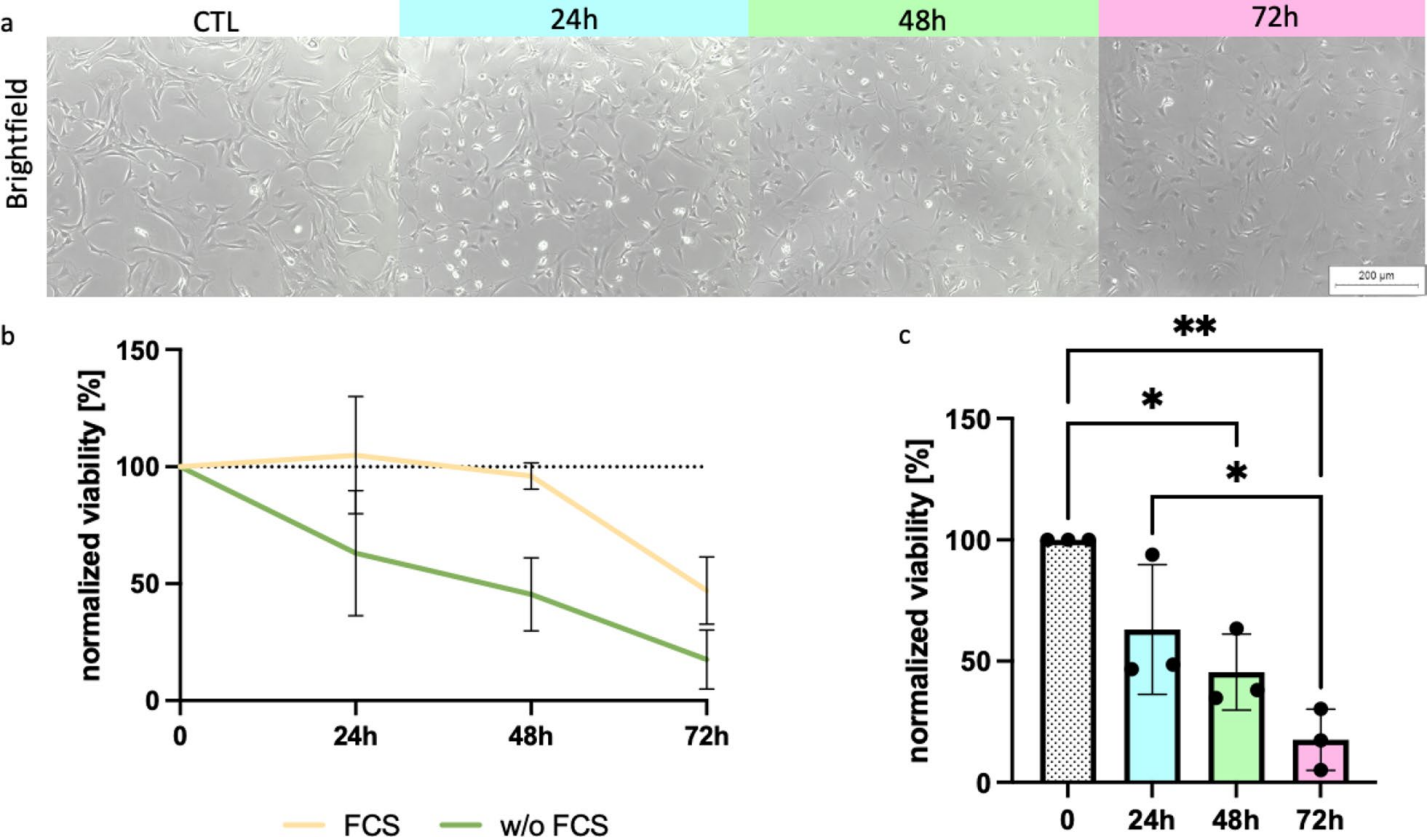
Viability and metabolic activity of AdSC cultured under serum-free conditions. Primary AdSCs (n = 3) were cultured without serum for 24h, 48h, and 72h. **a** Shows phase contrast micrographs of AdSCs of CTRL and after 24h, 48 h, and 72 h of serum-free culturing. **b** Displays the normalized viability of AdSC with (yellow line) and without serum (green line) (w/o FCS).The bar chart in **c** displays the comparison of cells cultured under serum-free conditions for 24h, 48h and 72h (n = 3, mean + SD) * p-value < 0.05; ** p-value < 0.01; ns: p-value > 0.05

The detrimental effects of serum-free conditions on MSCs are well described. [5, 14, 15] The viability and metabolic activity of AdSCs under FC- free conditions was determined by MTT assay. The AdSCs showed a decreased metabolic activity and viability compared to cells cultured in growth medium containing FCS (Fig. 2a–c). By taking the duration into account, viability decreased continuously reaching significancy after 48h of FCS-free conditioning. Moreover, cells exposed for 72h to serum-free conditions exhibited a significantly lower viability in comparison to cells cultured for 24h under same conditions (0h: 100% ± 0; 24h: 63.05% ± 26.76; 48h: 45.46% ± 15.70; 72h: 17.56% ± 12.64; p < 0.004).

### Quantification of sEVs reveals significant changes between different releasing periods

**Fig. 3:**
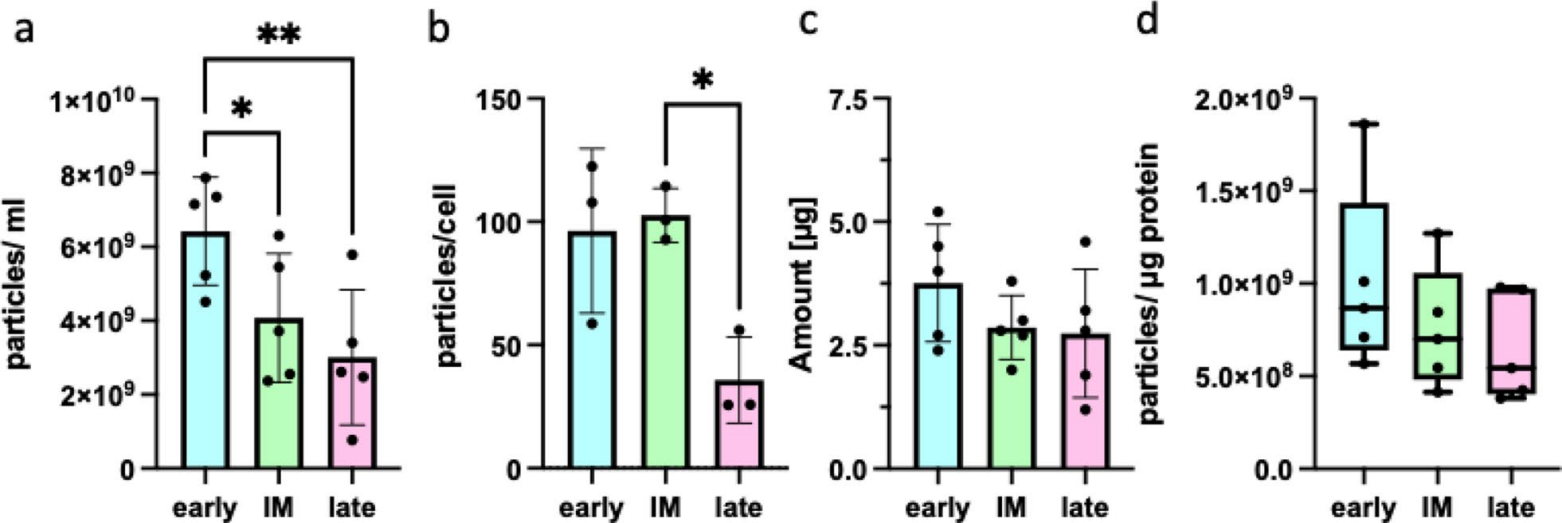
Quantification of sEVs. sEVs released in three-time windows were quantified by NTA in fluorescence mode, and micro-BCA protein assay for protein quantification. Histograms depict the mean ± SD with dots representing each single donor. **a** Particle quantification by NTA in fluorescence mode (n = 5). **b** Particles/cell ratio was calculated from particles measure in NTA fluorescence mode and respective viable cells at the end of conditioning period (n = 3). **c** Total yield amount of protein of sEV preparation per T175 flask (n = 5). **d** Particles per µg of protein were calculated from particles detected in NTA fluorescence mode and the respective amount of protein (n = 5). Whiskers displays the minimum and maximum range. The corpus gives the interquartile range with median inside the corpus. * p-value < 0.05; ** p-value < 0.01; ns: p-value > 0.05

Next, we determined the impact of serum-free conditioning on the quantity of sEVs secreted. sEVs were quantified with at least two methods microBCA kit for the amount of protein and via NTA for particle numbers. [17] The absolute number of detected particles decreased significantly in both, fluorescence (Fig. 3a) and scatter mode (figure not shown), under prolonged serum-free conditions. To evaluate, whether it is contributed to the absolute lower number of viable cells after 48h, and 72h, the particles-per-cell ratio was calculated. The sEV secretion remained stable over the first 48h and significantly dropped on the third day of serum-free culturing (Fig. 3b). The decreased total number of measured particles in NTA were sustained by the decreased total amount of protein, which was detected in the intermediate, and late group (early: 3.76 ± 1.19µg; intermediate: 2.86 ± 0.65µg; late: 2.74 ± 1.30µg; p > 0.05) (Fig. 3c). By calculating the particles-per-protein ratio we observed a higher amount of protein per particle in the intermediate, and late group (early: 1*10^9^ particles/µg ± 5.07*10^8^; intermediate: 7.55*10^8^ particles/µg ± 3.30*10^8^; late: 6.59*10^8^ particles/µg ± 2.93*10^8^; p > 0.05) (Fig. 3d). However, this trend was not statistically significant. Nevertheless, the observations are indicative of larger vesicles, like apoptotic bodies, or higher contamination with extravesicular proteins complexes and non-vesicular extracellular particles, e.g. cell debris [34–36].

### Duration of the conditioning period influences the size and phenotypic expression of sEVs

**Fig. 4:**
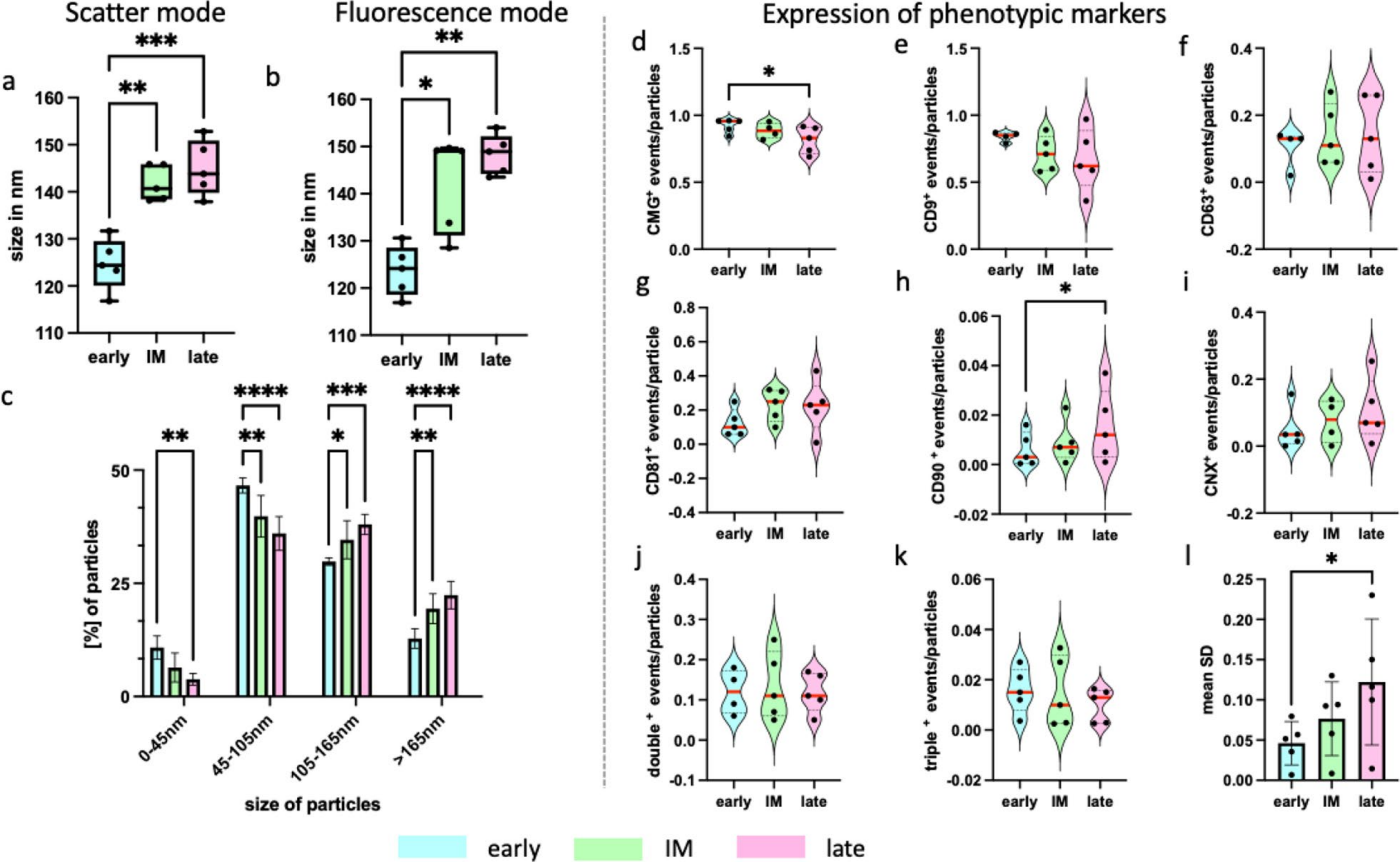
Size distribution and immunophenotype of sEVs. sEVs size distribution obtained by NTA in fluorescence mode and scatter mode. Histograms depicts the mean ± SD. Whiskers of boxplots display the minimum and maximum range. The corpus gives the interquartile range with a median inside the corpus. **a**, **b** Boxplots show the median size of particles in scatter mode and fluorescence mode as displayed, respectively. **c** Bar chart shows percentage inside each population of following size classes; exomere 0-45nm; small sEVs 45-105nm; large sEVs 105-165nm; > 165nm for particles larger. **d**–**i** Violin bars show ratio of CMG, CD9, CD63, CD81, Calnexin (CNX), CD90 positive events normalized on all detected particles determined by Imagestream IsX. **j**, **k** display the ratio of colocolized double or triple expressed tetraspanines (CD9, CD63 and CD81). **l** shows the mean standard deviation between single donors inside one EV preparation of all expression markers investigated before. n = 5; * p-value < 0.05; ** p-value < 0.01; *** p-value < 0.001; **** p-value < 0.0001; ns: p-value > 0.05

The size of the EVs was determined by NTA in scatter, and fluorescence mode in combination with a membrane stain. The median particle size was significantly smaller within first the 24h in comparison to those released during latter periods regardless of detection mode (fluorescence mode: early: 97.36 ± 2.39nm; intermediate: 110.8 ± 8.96nm; late: 118.1 ± 4.40nm; p < 0.05) (Fig. 4** a,b**). Superimposing a size-based classification revealed a heterogenous population of EVs, including exomeres (0-45nm), small sEVs (45-105nm), and large sEVs (105-165nm). [37, 38] Within the first 24h, the sEV production comprises significantly more exomeres, and small sEVs. After 24h, there is a shift in size distribution, and a higher number of large sEVs and bigger particles were quantified ** (**Fig. 4c).

Surface proteins used for characterization of sEVs include most established tetraspanines, like CD9, CD63, CD81, CD82 and are extended by negative markers such as calnexin (CNX), and cell type-specific markers—CD73,CD90, CD105- which are derived from parent cells. [39] The expression of these markers was analyzed on single vesicle level by imaging-based flow cytometry with Imagestream IsX. CMG as a marker for lipid membraned particles was significantly higher expressed in early and intermediate group than in late released sEVs (early: 0.92 ± 0.05; intermediate: 0.89 ± 0.06; late: 0.82 ± 0.10; p < 0.05). Most dominant expression in all groups was observed for CD9 (early: 0.84 ± 0.04; intermediate: 0.71 ± 0.13; late: 0.67 ± 0.23; p > 0.05) followed by CD81 (early 0.12 ± 0.08; 0.23 ± 0.09; late:0.22 ± 0.15; p > 0.05), and CD63 (early:0.11 ± 0.06; intermediate: 0.14 ± 0.09; late: 0.14 ± 0.12; p > 0.05) (Fig. 4 e–g). Further, vesicles were positive for CD90, demonstrating their descent from AdSCs (Fig. 4h). A contamination with particles positive for calnexin was observed in all groups (Fig. 4i). [40] In view of colocalization, about 11.8% to 13.4% of vesicles expressed more than one tetraspanine (Fig. 4j), and 1.0% to 1.6% were positive for all three of them (Fig. 4k). Except for CD90, the mean expression of the single markers did not differ significantly between the groups. Nevertheless, a donor-variance between the sEVs groups for a single marker expression was observed, which was confirmed by a statistically higher mean standard deviation of late sEVs in comparison to early, and intermediately released sEVs (Fig. 4l).

### Cryo-EM and AFM confirm findings of NTA, showing a high heterogeneity of vesicles regarding their size and membrane structure

**Fig. 5:**
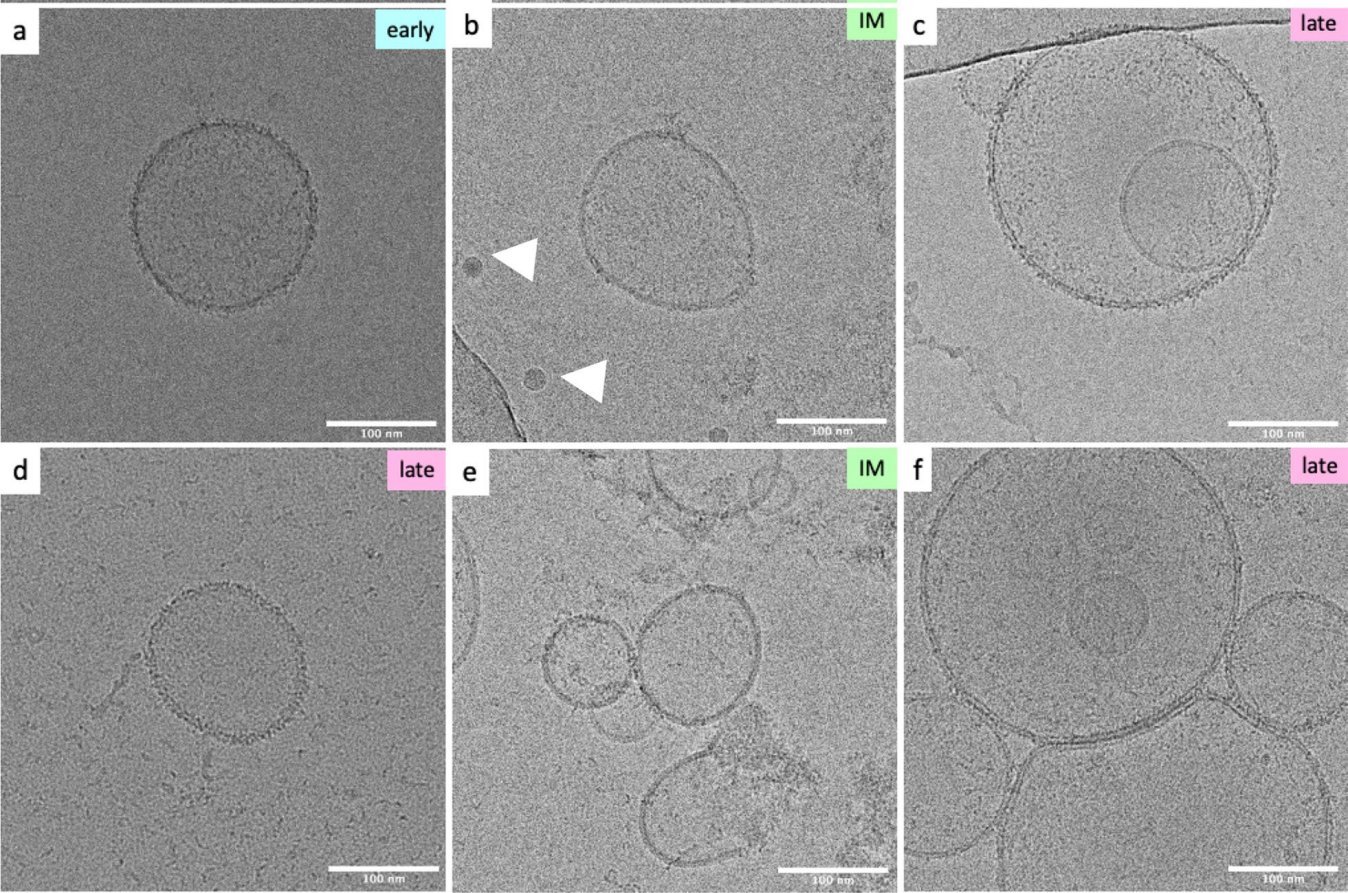
Exemplary micrographs of sEV obtained with cryo-electron microscopy. In the right corner the source of exemplary pictures is displayed—early, intermediate (IM), late released sEVs. To avoid wrong conclusions, we only display the exemplary character without any interpretation about the whole population found in one group. **a,d** A bilayer membrane vesicles around 120nm found in early, and late sEV preparation. **b** Shows size heterogeneity found in intermediately released sEVs with a 21nm sized vesicle, complying with a ribosome (white arrowhead). **c,f** Show a multilayer vesicle or one vesicle entrapped in a larger one. **e,f** Shows partially aggregated, multiple vesicles. n = 1

Cryo-EM pictures sustain our results from NTA, showing a heterogenous population of vesicles, including sEVs with a round shape and bilayer structure in all groups (Fig. 5a,b,d). In accordance with the literature, we found diverse populations of sEVs with various layer structures, like single (Fig. 5b, white arrow head), and double membraned (Fig. 5a-f) as well as double, and multilayered vesicles (Fig. 5c,e,f), which might be explained by sample preparation, immature or unprocessed EVs, or cell debris [41, 42]. Moreover, membraned particles in a size around or smaller than 50nm (Fig. 5b,c) were present in our preparations, which would comply with exomeres or ribosomes, respectively. Agglomerates of sEVs are well described in literature. We identified these formations (Fig. 5e,f), which might deliver a signal that may be detected by NTA as a bigger particle rather than smaller several particles. Further heterogeneity of vesicles could be observed by different electron densities visible through different gradients of grey, which is indicative of miscellaneous cargo. As these pictures represent single vesicles, only qualitative interpretations can be made.

**Fig. 6:**
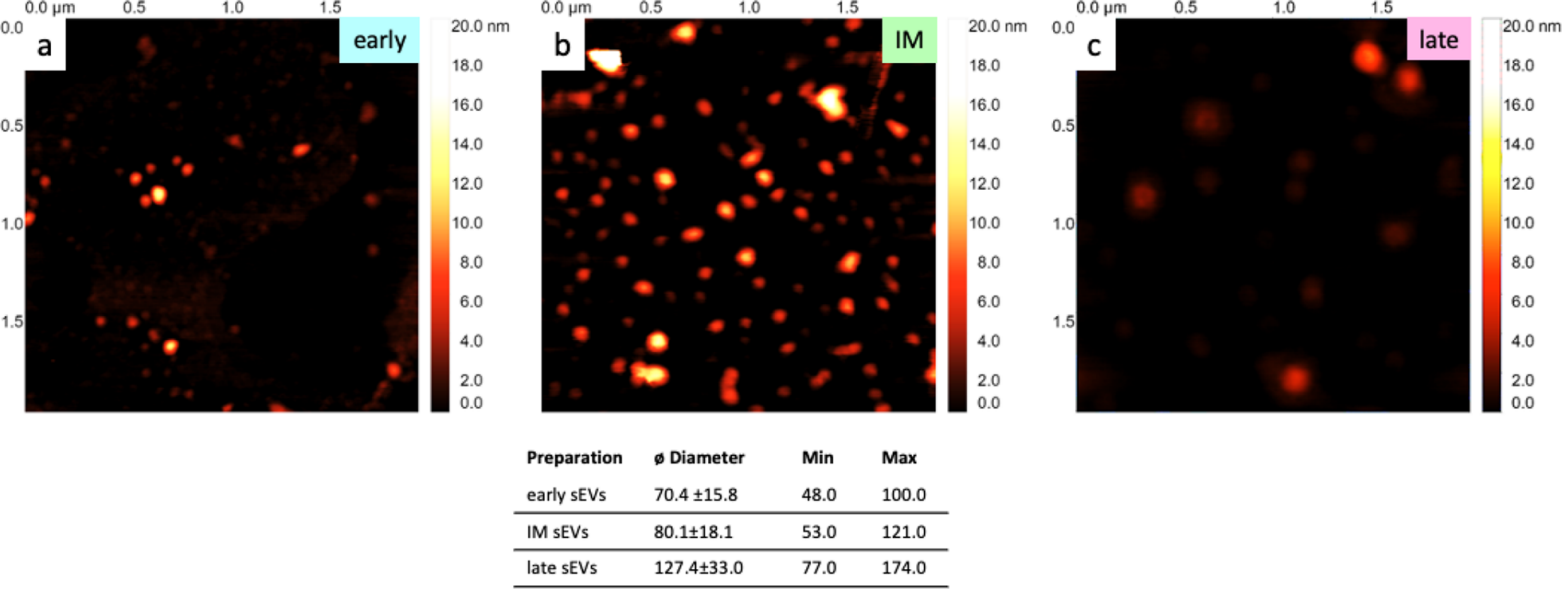
Atomic Force Microscopy of sEVs. Atomic Force Microscopy (AFM) pictures were taken of sEVs from early (**a**), intermediate (**b**) and late released (**c**) sEVs. All values are given in nm ± SD. n = 1

Subsequently to Cryo EM analysis, AFM measurements of the sEVs preparations were performed (Fig. 6a–c). The detected particles showed a positive correlation between diameter and serum-free conditioning time. The particles demonstrated in the early phase preparation the smallest mean size with lowest heterogeneity, followed by intermediate vesicles. Late-released sEVs were in the mean the largest particles, which could also explain the results obtained by NTA.

### 3.5. Principal component analysis reveals distinct populations of sEVs regarding their time window of release.

**Fig. 7:**
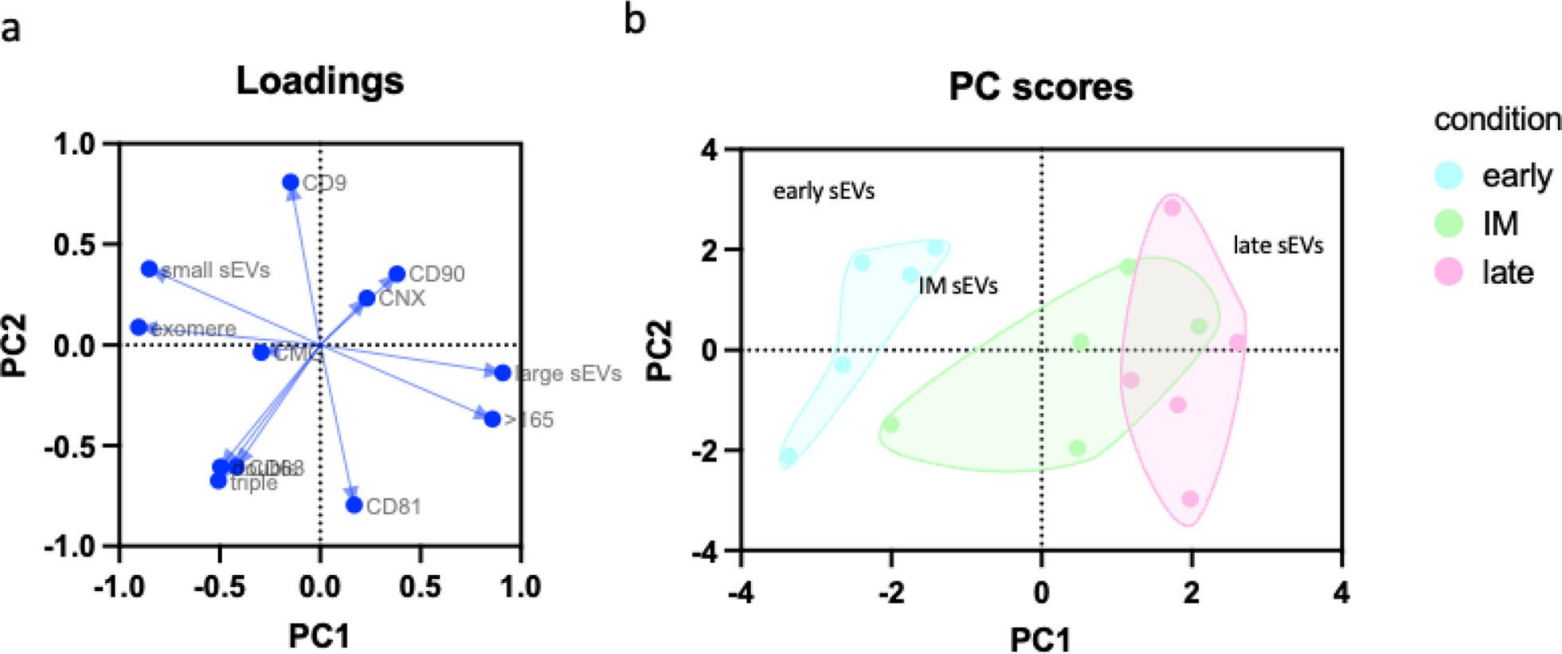
Principal Component Analysis (PCA) of sEVs preparations and cell lysates. **a** Shows the loadings of variables used for PCA. **b** Distribution of donors and time releasing periods of sEVs preparation. n = 5

The morphological and phenotypic characteristics were used to test for homogeneity regarding the size, and phenotype of sEVs between different donors. Variables loaded into principal component analysis (PCA) are displayed in Fig. 7a. In this analysis CD9^+^ particles are more associated with smaller size, whereas CD81^+^ demonstrated a higher correlation with larger vesicles. The population of early sEVs could be clearly segregated from those released in intermediate and late periods (Fig. 7b), where intersection between different donors could be observed. Moreover, taken the spatial expansion into consideration, the intermediate and late sEVs fraction were dispersed on a greater area, whereas the donors of early sEVs are spaced closest to each other, speaking of a higher homogeneity between different donors. Early sEVs correlated more with the size class of small sEVs and exomeres as wells as with a higher expression of CMG, CD63 and colocalization of two, and three tetraspanines. In contrast, late-released sEVs are associated with CD90, Calnexin, CD81 and larger particles.

### 3.6. Early sEVs significantly increase confluency and proliferation of primary Schwann cells, but not intermediate and late sEVs.

**Fig. 8:**
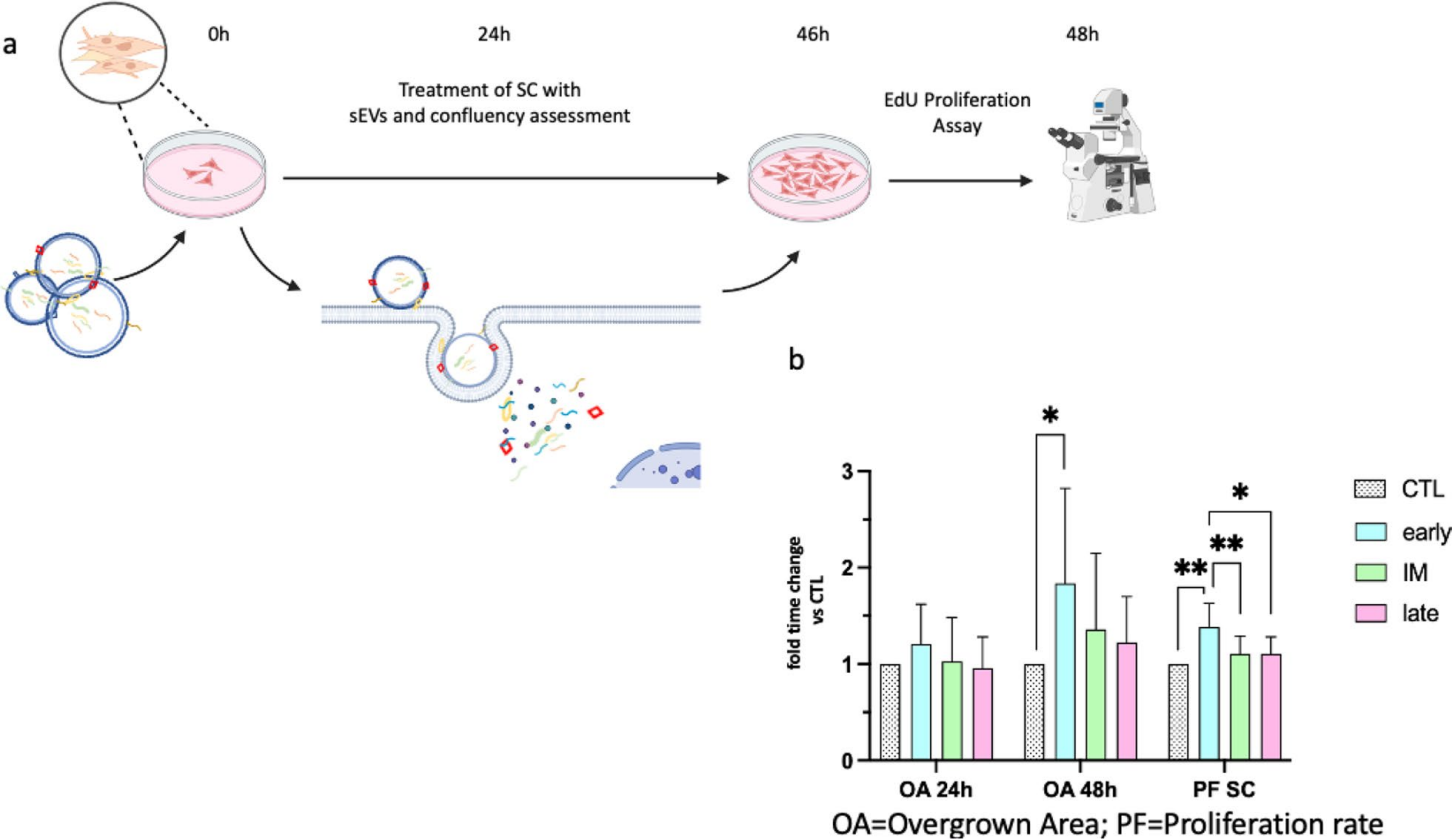
Proliferation experiment. **a** Timeline of proliferation assay. Created with BioRender.com **b** Bar charts display the mean, whiskers display SD. All rates were normalized on control group (PBS only). Confluency (overgrown area/OA) shows the increase of overgrown area with primary Schwann cells (n = 5) measured after 24h and 48h of coculture. The proliferation rate (PF) of SCs was calculated by EdU positive/ (Sox-10 & DAPI) positive cells after 48h of coculture. For better clarity,” non-significant” labels were edited out. * p-value < 0.05; ** p-value < 0.01

sEVs derived from MSCs show proliferative effects on diverse cell types. [4, 6, 12, 13, 43] Subsequently, to the characterization of sEVs, we investigated the proliferative properties of sEVs derived from AdSCs in coculture with SCs (Fig. 8a). The presence of primary SCs was demonstrated by the expression of S-100, and SOX-10. CD90 was used as a fibroblast marker (supp. Figure 4a–e). The purity was quantitatively assessed by the ratio of SOX-10^+^ to DAPI^+^ cell nuclei (supp. Figure 4f–k). All donors showed a comparable purity (p = 0.76).

The proliferative potential was assessed by an increase of confluency as well as with a proliferation assay. Brightfield pictures were acquired at the beginning of the experiment, 24h and 48h after cocultivation and the overgrown area was calculated as described methods section. An increase in confluence after 24h could be only observed in Schwann cells treated with early, and IM released sEVs (Fig. 8b). After 48h a significant increase was achieved only by sEVs from the early group (CTL: 1.39 ± 0.84; early: 2.88 ± 0.80; IM: 1.90 ± 0.89; late: 1.66 ± 0.38; p < 0.05). The SC-specific proliferation rate was measured by the ratio of SOX-10 positive cells to EdU. In concordance with confluency results, SCs treated with early sEVs proliferated significantly higher as compared with all other groups (fold time increase of proliferation in comparison to control: early: 1.38 ± 0.24; p = 0.002; IM: 1.05 ± 0.19; p = 0.55; late: 1.04 ± 0.18; p = 0.56).

## Discussion

The body of literature on EVs has expanded over the several past years. The important therapeutic potential of sEVs derived from MSCs is becoming well-established, providing promising prospects for clinical use in the broad field of regenerative medicine [3, 4]. Nevertheless, EVs are a heterogenous group of vesicles released by cells [42] and many factors were identified to exert an influence on their morphological, and functional properties making their use difficult and problematic with special emphasis on a clinical use [13, 44]. For this reason, the International Society for Extracellular Vesicles (ISEV) releases intermittent guidelines to minimize variations in results thereby creating a foundation for standardized, reproducible, and comparable research [17, 21]. Fetal calf serum (FCS) contains albumin, growth factors, vitamins, minerals, electrolytes and EVs [20, 45]. To avoid a contamination with exogenous EVs found in serum (e.g. FCS or human platelet lysate) [14, 20], the conditioning period under serum deprivation is a critical step during the preparation of sEVs from cell culture. We observed a reduced cell metabolic activity and viability of primary AdSCs under FCS deprivation in comparison to growth medium containing FCS. Zhu et al. (2006) described a caspase-dependent apoptosis of AdSC as a reaction on hypoxia and serum deprivation [46]. EV-depleted serum or chemically defined serum-free media may be valuable alternatives to overcome this issue. In EV-depleted FCS Eitan et al. (2015) found a lower viability of human U87 glioblastoma cells in comparison to common FCS [14]. Cells cultured in chemically defined serum-free media demonstrated a comparable viability in comparison to FCS containing medium [47, 48]. Thus, commercial serum replacement products could be an alternative to serum deprivation conditioning [21, 48].

EVs reflect the condition of their parent cells and the characteristics of sEVs preparations may be influenced by the effects on the cultured cells by different media composition [16, 49]. Current available data regarding the conditioning period under serum deprivations is scarce and no recommendations or regulations have as yet been established by the ISEV. Variables analyzed so far include the size and quantity of released sEVs, revealing an increase in size with EV collection from longer culture times [50, 51]. Furthermore, Oesterreicher et al. (2020) suggested a higher contamination with cell debris from cells conditioned for 48h as compared with a 24h lasting conditioning period [52]. They postulated that the population of sEVs differs in relation to time by comparing sEVs released at 24h and 48h from the same cultures. However, the EV populations released in distinct time frames were not separately analyzed, but the difference between 24 h and the collective EV population over 48 h was examined.

Our approach of sequential collection and refreshment of conditioned medium each 24h enabled us to receive distinctly produced sEVs populations in different time windows. Further, we compared these populations by quantification, size distribution, phenotypic expression, and their proliferation potency. Our results revealed distinct populations of sEVs, which all met common sEVs characteristics [17, 53, 54]. There is growing evidence that sEV populations of various sizes possess a unique composition of proteins [16, 55, 56] and other cargos [37] and effect on gene expression (2). By applying this size-classification, we could demonstrate, that in different time windows AdSCs release a significantly changed composition of particles under cellular stress, which comply with exomeres (smaller than 50nm), small (50-100nm), and large sEVs (100 to 160nm). In particular, AdSCs released smaller vesicles in the early conditioning period, whereas a higher number of larger particles were detected after longer serum-free conditioning of the cells. These particles might as well correspond to cellular debris, immature vesicles or larger apoptotic bodies [36]. Taking the results of cryo-EM into consideration, we observed an increased incidence of multilayered vesicles in the intermediate and late fraction. Whether this phenomenon is caused by an increased stress stimulus, as hypothesized by Broad et al. (2023), needs further investigation [57].

Fundamental consensus for tetraspanins exists in their enrichment on sEVs. CD63 is more present in the endosomal region, whereas CD81, and CD9 are more abundant in the plasma membrane, which might also contribute to certain functional properties [38] [55, 58–60]. The enhanced release of CD9^+^ sEVs in the initial phase correlates well with its early endocytic origin as a first response of cells to stress due to serum deprivation [39]. Noteworthy, in studies which exerted hypoxic [61], mechanical stress [62] or deprivation of glucose [63] showed equivalent observations. Moreover, van de Wakker (2024) described a different distribution of tetraspanins in correlation with the size of particles. CD81 was more prevalent in larger particles, whereas CD63 and CD9 was found to be enriched in smaller particles [34]. Nevertheless, recently released publications focusing on size-dependent distribution of tetraspanins found variant results [37, 38, 64]. Some authors suggested, that it might be caused by different cell types, isolation methods and unknown parameters [37, 49, 56].

sEVs derived from primary MSCs, like AdSCs, are well described to exert an effect on proliferation and regeneration in various cell types and injury models. One established application for sEV in regeneration is a co-culture with Schwann cells [6, 11–13, 24]. Whereas some modification may improve the therapeutic potential and outcome after sEV application [1], it is generally of high interest to eliminate all methodical inconsistencies for the sake of reproducibility [4]. Prolonging the conditioning period in order to increase the yield is common and there is a lack of data about the downstream effects. Our proliferation experiments performed with sEVs separated by their releasing period revealed significant differences in their proliferative potency. Only sEVs released within the first period showed the well-known proliferative effects on SCs, whereas the regenerative potency was significantly lower in sEVs produced later than 24h. A previously published experiment demonstrated a potential correlation between smaller particles and a higher proliferative capacity [34]. In our study, we observed a significantly smaller population of EVs in the early group, which might correlate with the superior proliferative activity. However, there are limitations to this study. The primary aim of this work was to highlight issues associated with serum starvation conditioning as a common practice during EV preparation. This manuscript emphasizes that increasing particle yield by extending the serum starvation period should be critically evaluated, as it may influence the bioactive capacities of released particle. Nevertheless, cellular processes, compositional analysis EV, their release mechanisms, and EV cargo were not addressed in this study. Future investigations should aim to analyze EVs generated under different conditioning periods using lipidomic, proteomics, or RNA sequencing approaches to gain deeper insights into their composition and cargo.

## Conclusion

In conclusion, we present a multiplexed comparative analysis of sEVs derived from AdSCs separated by their temporal release window. Most studies on sEVs report the conditioning period and the media used. Our results provide additional information about the optimal conditioning period window under serum-free conditions. In accordance with common characterization criteria for EVs, particles in all groups met the characterization criteria for sEVs [17]. However, our data suggest the use of sEVs released in the first 24 h for optimal proliferation of Schwann cells, an important cell type in peripheral nerve regeneration. We observed a highly coherent, and more homogenous population of particles with the well described prolific effect with application of sEVs produced by AdSCs at 24 h compared to those from later time windows. However, we only evaluated the effects of sEVs on proliferation of Schwann cells. Future experiments will be necessary to determine if these results are generalizable to other cell types. Moreover, it will be important to investigate the cargo composition of EVs. The results clearly indicate that the conditioning period is a highly influencing variable which requires a standardization for comparative effects of sEVs on cellular interactions.

## Supplementary Information

Supplementary material 1: Supplementary Table 1: Antibody and Dye list. Supplementary Figure 1: Gating strategy for Imagestream. a-b shows plot of Channel 02(Ch02- 488nm) and Channel 06 (Ch06 - scatter). a shows results of unstained sEVs (blue) and beads fraction (red). b displays results of sEVs stained for CMG (488nm). In deduction, a gate was set for Ch06 =0 (“no beads”) for the sEV fraction displayed in c. d-e shows histograms of stained sEVs signals in Ch02 (488nm) and Ch05 (594nm) gated for the “no beads”, which equals sEV fraction. Particles positive for CD9 or CD63 were all signals defined equal to 0 (“no beads”) and larger than 0 in the respective detection channel. f displays exemplary a triple positive event. Supplementary Figure 2: Immunophenotypic characterization of primary AdSCs. a shows the nuclei stained with DAPI. b-d shows the expression of CD73, CD90 and CD105, respectively. e shows the merged micrograph. Supplementary Figure 3: Microscopy images of AdSCs culture in p3 in growth medium and after differentiation. a shows AdSCs in common growth medium. b-d shows AdSCs after differentiation. b,c demonstrates presence of adipocytes and osteocytes after 21 day of culture and stain with Oil Red O and Alizarin Red, respective. 3d shows chondrogenic pellet mass positive for Alcain blue. Supplementary Figure 4: Immunophenotypic micrographs of primary Schwann cell cultures. a-e Show exemplary confocal image in a 20-fold magnification of primary Schwann cell culture with staining for S-100, CD90, DAPI, Vimentin. a Merged picture of all channels. b Nuclei staining. c S-100 positive cells, which is a common Schwann cell marker. d Shows CD90 positive cells, which comply with fibroblasts. e Vimentin is a cytoskeletal protein demonstrates presence of cells. f-i shows exemplary micrographs from staining panel for proliferation assay with DAPI (g), SOX-10, a common Schwann cell nuclei staining (h), and EdU for cells in S-phase (i). j-k Bar charts gives the purity rate by the calculation of SOX-10/DAPI positive cells by donor (n=5) (j) and treatment group (n=5) (k). All scale bars in a-i represent 100µm. Upper border of bar charts gives the arithmetic mean. Whiskers show the SD. A dot represents a single donor. ns: p-value>0.05. Supplementary Figure 5: Processing of brightfield pictures for calculation of overgrown area. Brightfield pictures were obtained over 48hrs of coculture of Schwann cells with sEVs or vehicle control at the same marked location Brightfield pictures were processed with Fiji software to calculate the overgrown area by Schwann cells. a shows the brightfield pictures and b the processed pictures used for overgrown area calculations.

## Data Availability

No datasets were generated or analysed during the current study.

## Abbreviations

AdSCs: Adipose tissue-derived stromal cells
CM: Conditioned medium
CNX: Calnexin
EV: Extracellular vesicle
FCS: Fetal calf serum
HBSS: Hanks balanced salt solution
IM: Intermediate
ISEV: International Society for Extracellular Vesicle
MSC: Mesenchymal stromal cells
NTA: Nanoparticle tracking analysis
PCA: Principal component analysis
PBS: Phosphate buffered saline
SC: Schwann cells
sEV: Small extracellular vesicles
