# Correction: Conditioning period impacts the morphology and proliferative effect of extracellular vesicles derived from rat adipose tissue derived stromal cell

**DOI:** 10.1186/s12951-025-03345-7

**Published:** 2025-04-02

**Authors:** Anton Borger, Maximilian Haertinger, Flavia Millesi, Lorenz Semmler, Paul Supper, Sarah Stadlmayr, Anda Rad, Christine Radtke

**Affiliations:** 1 Department of Plastic, Reconstructive and Aesthetic Surgery, Medical University of Vienna, Währinger Gürtel 18-20, 1090 Vienna, Austria; 2 Austrian Cluster for Tissue Regeneration, Vienna, Austria

**Correction to: Journal of Nanobiotechnology (2025) 23:164**

10.1186/s12951-025-03273-6

Following publication of the original article, it was reported that there was an error in the authorship list that the first name and the surname were swapped of all authors.

The incorrect author names are Borger Anton, Haertinger Maximilian, Millesi Flavia, Semmler Lorenz, Supper Paul, Stadlmayr Sarah, Rad Anda and Radtke Christine.

The correct author names are Anton Borger, Maximilian Haertinger, Flavia Millesi, Lorenz Semmler, Paul Supper, Sarah Stadlmayr, Anda Rad and Christine Radtke.

The author group has been updated above and the original article has been corrected.

