# Supplementary material for: Conditioning period impacts the morphology and proliferative effect of extracellular vesicles derived from rat adipose tissue derived stromal cell
Source: J Nanobiotechnology. 2025 Mar 4;23:164. doi: 10.1186/s12951-025-03273-6 (PMC11877948; doi:10.1186/s12951-025-03273-6)
Supplement: Supplementary file 1 — Supplementary material 1: Supplementary Table 1: Antibody and Dye list. Supplementary Figure 1: Gating strategy for Imagestream. a-b shows plot of Channel 02(Ch02- 488nm) and Channel 06 (Ch06 - scatter). a shows results of unstained sEVs (blue) and beads fraction (red). b displays results of sEVs stained for CMG (488nm). In deduction, a gate was set for Ch06 =0 (“no beads”) for the sEV fraction displayed in c. d-e shows histograms of stained sEVs signals in Ch02 (488nm) and Ch05 (594nm) gated for the “no beads”, which equals sEV fraction. Particles positive for CD9 or CD63 were all signals defined equal to 0 (“no beads”) and larger than 0 in the respective detection channel. f displays exemplary a triple positive event. Supplementary Figure 2: Immunophenotypic characterization of primary AdSCs. a shows the nuclei stained with DAPI. b-d shows the expression of CD73, CD90 and CD105, respectively. e shows the merged micrograph. Supplementary Figure 3: Microscopy images of AdSCs culture in p3 in growth medium and after differentiation. a shows AdSCs in common growth medium. b-d shows AdSCs after differentiation. b,c demonstrates presence of adipocytes and osteocytes after 21 day of culture and stain with Oil Red O and Alizarin Red, respective. 3d shows chondrogenic pellet mass positive for Alcain blue. Supplementary Figure 4: Immunophenotypic micrographs of primary Schwann cell cultures. a-e Show exemplary confocal image in a 20-fold magnification of primary Schwann cell culture with staining for S-100, CD90, DAPI, Vimentin. a Merged picture of all channels. b Nuclei staining. c S-100 positive cells, which is a common Schwann cell marker. d Shows CD90 positive cells, which comply with fibroblasts. e Vimentin is a cytoskeletal protein demonstrates presence of cells. f-i shows exemplary micrographs from staining panel for proliferation assay with DAPI (g), SOX-10, a common Schwann cell nuclei staining (h), and EdU for cells in S-phase (i). j-k Bar charts gives the purity [file 12951_2025_3273_MOESM1_ESM.docx]

# Supplementary

**Supplementary Table 1: Antibody and Dye list**

|  | **Manufacturer** | **Product No.** | **Dilution** | **reactivity** | **host** |
| --- | --- | --- | --- | --- | --- |
| **Primary** |  |  |  |  |  |
| CD73 | Cell Signaling | D7F9A | 1:400 | - | rabbit |
| CD90 | Santa Cruz | sc-53116 | 1:400 | - | mouse |
| CD105 | R&D Systems | AF6440 | 1:400 | - | goat |
| S-100 | DAKO | Z0311 | 1:400 | - | rabbit |
| SOX-10 | Santa Cruz | sc-365692 | 1:100 | - | mouse |
| Vimentin | Themo Fisher | PA1-10003 | 1:400 | - | chicken |
| **Secondary** |  |  |  |  |  |
| AF-488 | Invitrogen | A32731 | 1:400 | rabbit | goat |
| AF-488 | Thermo Fisher | A-11029 | 1:200*/1:400 | mouse | goat |
| AF 594 | Thermo Fisher | A-21203 | 1:400 | goat | donkey |
| AF 647 | Thermo Fisher | A-21236 | 1:400 | mouse | goat |
| DyLight 650 | Thermo Fisher | SA5-10073 | 1:400 | chicken | goat |
| **Antibodies Conjugated** |  |  |  |  |  |
| CD9-AF488 | Santa Cruz | sc-13118 | 1:50 |  | mouse |
| CD63- AF594 | Santa Cruz | Sc-5275 | 1:50 |  | mouse |
| CD81- VioBlue | Miltenyi | 130-102-633 | 1:50 |  | hamster |
| Calnexin- AF647 | Santa Cruz | Sc46669 | 1:50 |  |  |
| CD90 - VioBlue | Miltenyi | 130-112-689 | 1:50 |  | human |
| **Other stainings** |  |  |  |  |  |
| CellMask Green | Thermo Fisher | C37608 | 1:500**/1:2000 | - | - |
| DAPI | Thermo Fisher | 62248 | 1:500 | - | - |

*for SOX-10 a higher concentration was used

** used for NTA


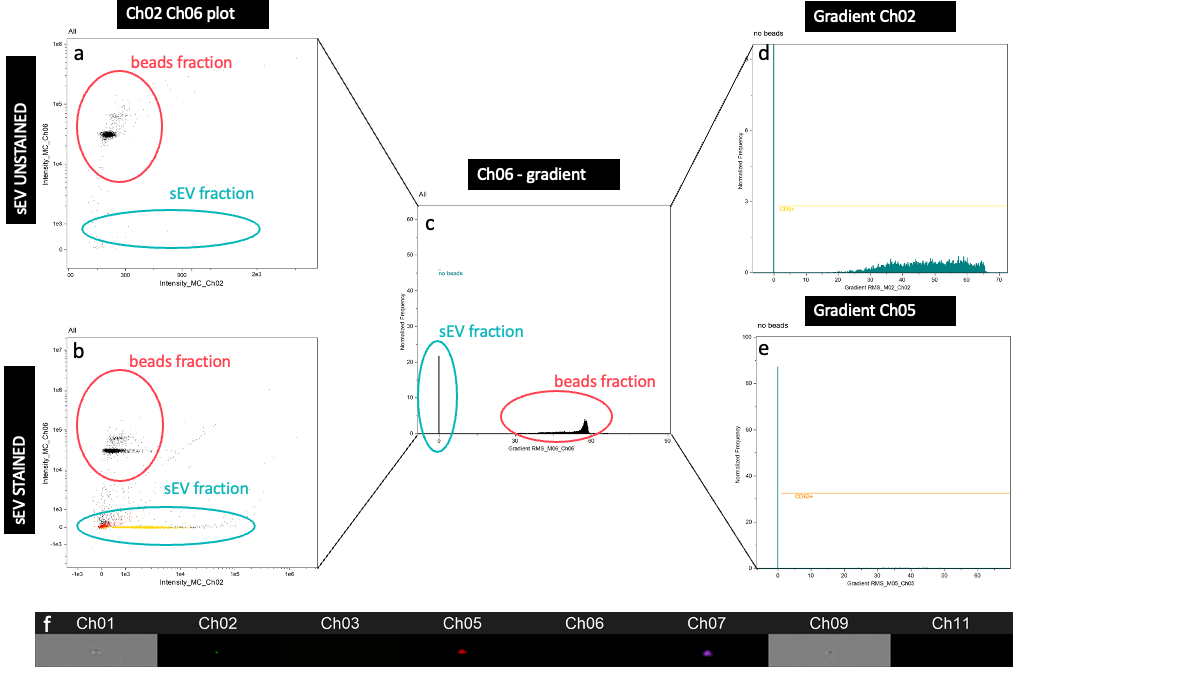


**Supplementary Figure 1: Gating strategy for Imagestream. a-b** shows plot of Channel 02(Ch02- 488nm) and Channel 06 (Ch06 - scatter). **a** shows results of unstained sEVs (blue) and beads fraction (red). **b** displays results of sEVs stained for CMG (488nm). In deduction, a gate was set for Ch06 =0 (“**no beads**”) for the sEV fraction displayed in **c**. **d-e** shows histograms of stained sEVs signals in Ch02 (488nm) and Ch05 (594nm) gated for the “**no beads**”, which equals sEV fraction. Particles positive for CD9 or CD63 were all signals defined equal to 0 (“no beads”) and larger than 0 in the respective detection channel. **f** displays exemplary a triple positive event.

**
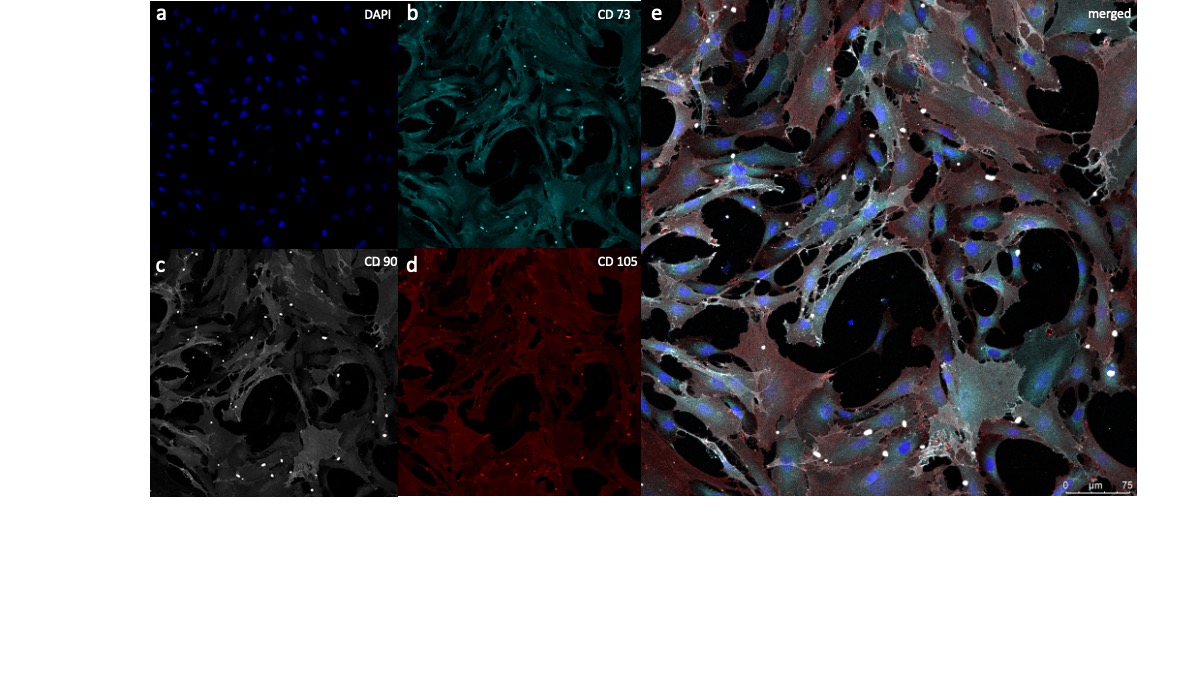
Supplementary Figure 2:** **Immunophenotypic characterization of primary AdSCs**. **a** shows the nuclei stained with DAPI. **b-d** shows the expression of CD73, CD90 and CD105, respectively. **e** shows the merged micrograph.


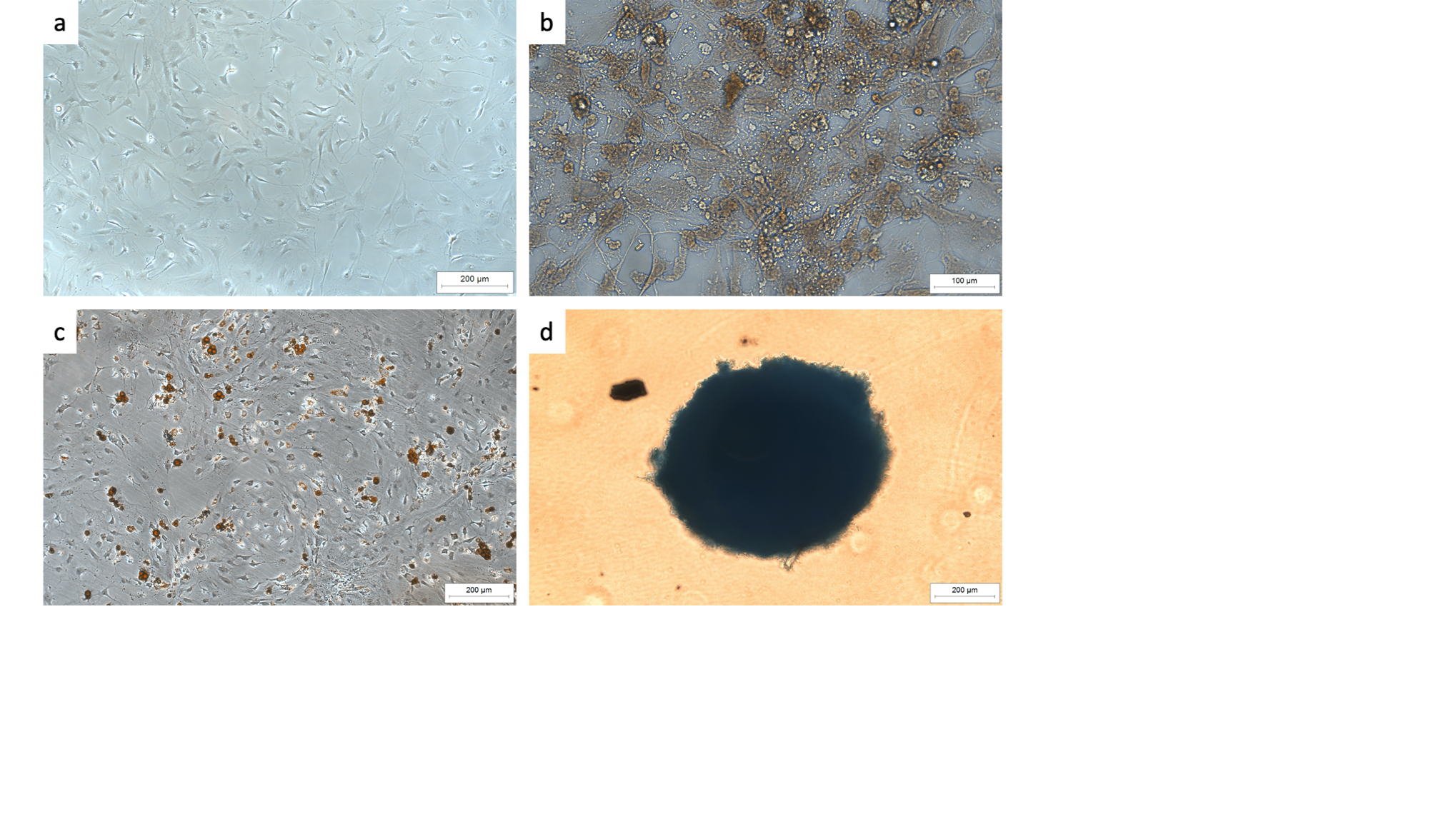


**Supplementary Figure 3: Microscopy images of AdSCs culture in p3 in growth medium and after differentiation**. **a** shows AdSCs in common growth medium. **b-d** shows AdSCs after differentiation. **b,c** demonstrates presence of adipocytes and osteocytes after 21 day of culture and stain with Oil Red O and Alizarin Red, respective. **3d** shows chondrogenic pellet mass positive for Alcain blue.


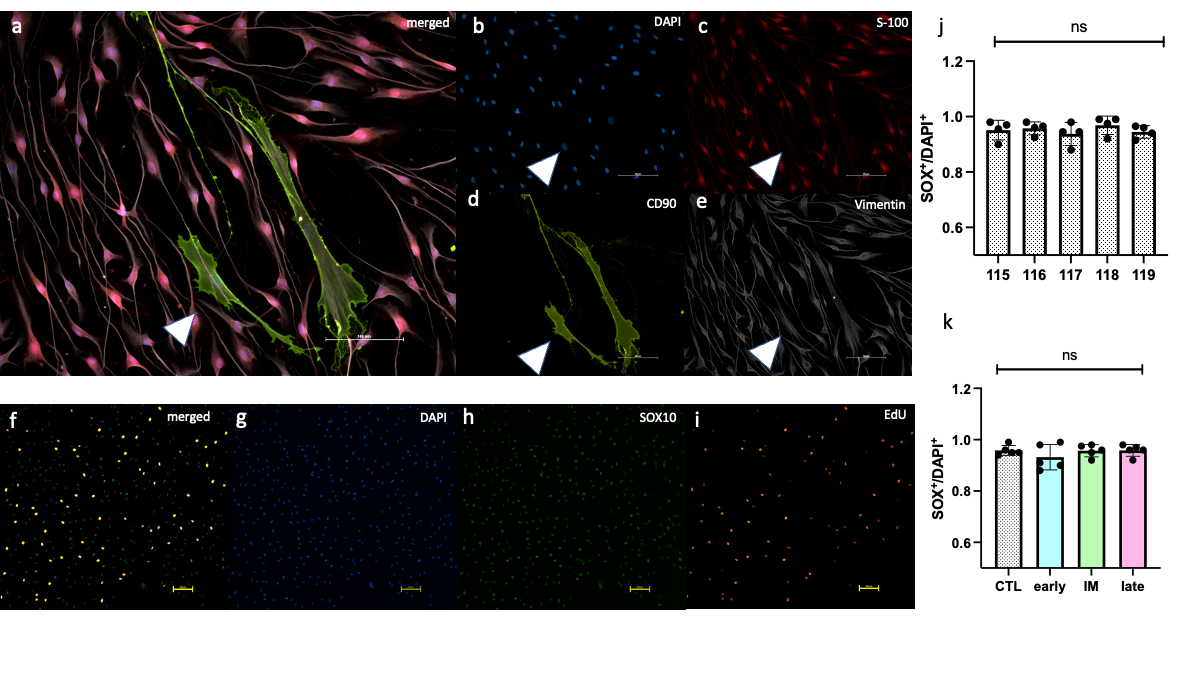


**Supplementary Figure 4: Immunophenotypic micrographs of primary Schwann cell cultures.** **a-e** Show exemplary confocal image in a 20-fold magnification of primary Schwann cell culture with staining for S-100, CD90, DAPI, Vimentin. **a** Merged picture of all channels. **b** Nuclei staining. **c** S-100 positive cells, which is a common Schwann cell marker. **d** Shows CD90 positive cells, which comply with fibroblasts. **e** Vimentin is a cytoskeletal protein demonstrates presence of cells. **f-i** shows exemplary micrographs from staining panel for proliferation assay with DAPI (**g**), SOX-10, a common Schwann cell nuclei staining (**h**), and EdU for cells in S-phase (**i**). **j-k** Bar charts gives the purity rate by the calculation of SOX-10/DAPI positive cells by donor (n=5) (**j**) and treatment group (n=5) (**k**). All scale bars in **a-i** represent 100µm. Upper border of bar charts gives the arithmetic mean. Whiskers show the SD. A dot represents a single donor. ns: p-value>0.05.


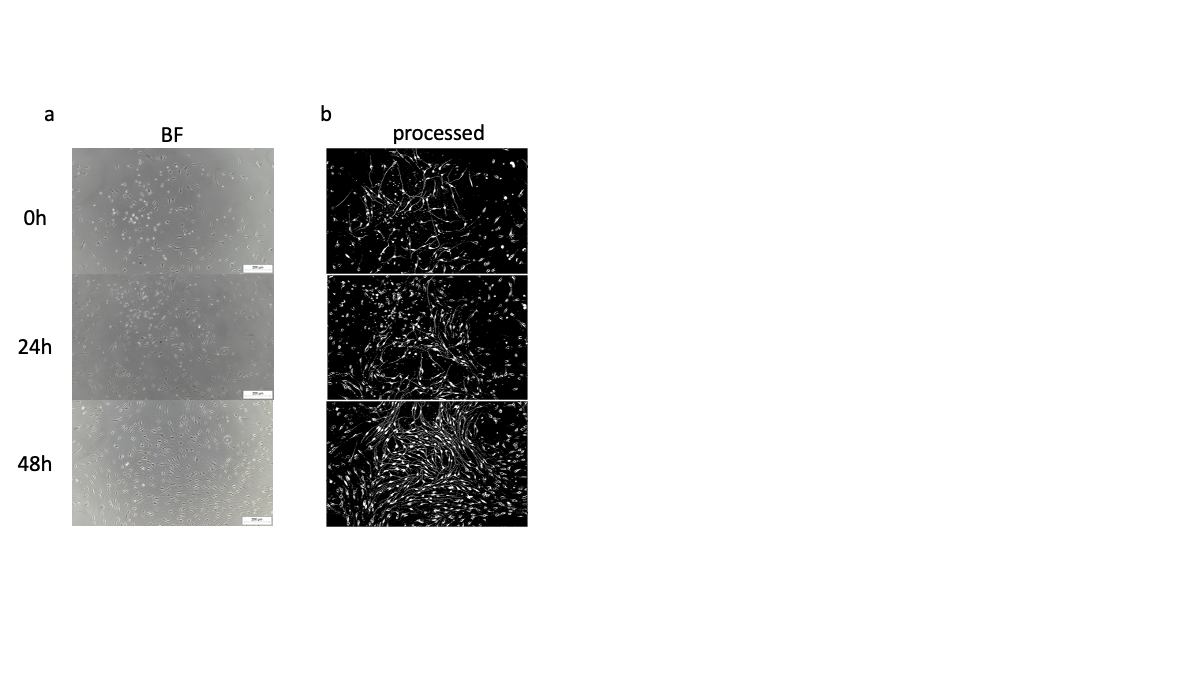


**Supplementary Figure 5: Processing of brightfield pictures for calculation of overgrown area.** Brightfield pictures were obtained over 48hrs of coculture of Schwann cells with sEVs or vehicle control at the same marked location Brightfield pictures were processed with Fiji software to calculate the overgrown area by Schwann cells. **a** shows the brightfield pictures and **b** the processed pictures used for overgrown area calculations.
